# Rootstock-Dependent Response of Hass Avocado to Salt Stress

**DOI:** 10.3390/plants10081672

**Published:** 2021-08-13

**Authors:** Silit Lazare, Yafit Cohen, Eitan Goldshtein, Uri Yermiyahu, Alon Ben-Gal, Arnon Dag

**Affiliations:** 1 Gilat Research Center, Agricultural Research Organization, Volcani Institute, M.P. Negev, Gilat 8528000, Israel; uri4@volcani.agri.gov.il (U.Y.); bengal@volcani.agri.gov.il (A.B.-G.); arnondag@volcani.agri.gov.il (A.D.); 2 Institute of Agricultural Engineering, Agricultural Research Organization, Volcani Institute, Rishon LeZion 7505101, Israel; yafitush@volcani.agri.gov.il (Y.C.); eitan@volcani.agri.gov.il (E.G.)

**Keywords:** crop trees, NaCl, remote sensing, salt susceptibility, trichomes

## Abstract

Salt stress is a major limiting factor in avocado (*Persea americana*) cultivation, exacerbated by global trends towards scarcity of high-quality water for irrigation. Israeli avocado orchards have been irrigated with relatively high-salinity recycled municipal wastewater for over three decades, over which time rootstocks were selected for salt-tolerance. This study’s objective was to evaluate the physiological salt response of avocado as a function of the rootstock. We irrigated fruit-bearing ‘Hass’ trees grafted on 20 different local and introduced rootstocks with water high in salts (electrical conductivity of 1.4–1.5 dS/m). The selected rootstocks represent a wide range of genetic backgrounds, propagation methods, and horticultural characteristics. We investigated tree physiology and development during two years of salt exposure by measuring Cl and Na leaf concentrations, leaf osmolality, visible damages, trunk circumference, LAI, CO_2_ assimilation, stomatal conductance, spectral reflectance, stem water potential, trichomes density, and yield. We found a significant effect of the rootstocks on stress indicators, vegetative and reproductive development, leaf morphogenesis and photosynthesis rates. The most salt-sensitive rootstocks were VC 840, Dusa, and VC 802, while the least sensitive were VC 159, VC 140, and VC 152. We conclude that the rootstock strongly influences avocado tree response to salinity exposure in terms of physiology, anatomy, and development.

## 1. Introduction

Commercial modern fruit orchards are typically composed of trees with scions grafted on to rootstocks of different genetic origins. Grafting is a major element of propagation processes and rootstock selection is a key component in successful and healthy orchard establishment [1]. Rootstock selection can be based on adaptability to soil characteristics, pest and/or disease pressure, and desired root–scion propagation and growth interactions. Avocado (*Persea americana*) is a crop of rising importance, with high nutritional and economic values [2]. The expansion of avocado cultivation areas might be challenging due to the crop’s susceptibility to stress brought upon by weather, irrigation water quality, and soil conditions including temperature, acidity, oxygen availability, and salinity [3,4,5,6]. The sensitivity of avocado to such conditions highlights that trees must be conditioned and adjusted to their specific local environment. The complexity in advancing its production increases when considering that avocado consumers generally prefer a specific variety (‘Hass’) [7]. Rootstocks must therefore be matched to cultivation conditions and rootstock–scion relationships and subsequent crop performance should be evaluated. Rootstocks have long been used to help sensitive species cope with biotic and abiotic stresses. With avocado, most research has focused on diseases such as phytophthora root rot [8], botryosphaeria branch dieback [9], and verticillium wilt [10]. Abiotic stresses addressed in rootstock studies include drought [11], calcareous soil [12], and salinity [13].

Salt susceptibility in agricultural crops is defined by a reduction in plant growth, development, and productivity [14]. Avocado is considered one of the most salt-sensitive crops [15]. Its rootstocks are of three primary genetic sources: Mexican, West Indian, and Guatemalan [16]. Mexican rootstocks were found to be more susceptible to salinity than others [17,18]. Physiological responses of avocado to salt exposure or wastewater irrigation were investigated and reviewed during the last 15 years in a number of studies [6,19,20,21]. The studies focused mostly on scion photosynthesis rates, productivity, and nutritional status, revealing the negative effect of salinity exposure on these indices. In our opinion, the influence of rootstocks on avocado response to salinity is particularly engaging, and we therefore invested in data collection from many rootstocks grafted with the same (Hass) scion, in order to identify their distinct effects.

Salt stress is known to negatively affect growth and productivity through both osmotic and toxic mechanisms [22]. The osmotic damage is expressed as changes in water status, similar to that of drought, measured by reduction in stem water potential (SWP) [23,24]. The potential refers to the energy status of water in a plant, which reflects the balance between water status in the soil, water taken up from soil by roots, atmospheric water status and demand, and plant physiological responses, particularly stomatal resistance or closure [25]. The root’s water uptake is highly influenced by exposure to salts, which may change root hydraulic characteristics and decrease their function [26,27]. Salt exposure decreases water potential and consequently inhibits a number of metabolic and physiological functions. To compensate for salinity induced stress, some plants respond through osmotic adjustment [28]. This mechanism includes an active gradual decrease in cellular water content, which leads to accumulation of solutes within the cell and water movement inside it [29]. Osmotic adjustment develops gradually under slow and uniform rates of dehydration. With this drought-acclimation mechanism, plants maintain high turgor potential under dehydration and avoid inhibition of their functions. Under drought stress, high solute concentrations may indicate tolerance, but when salt stress causes osmotic instability, high leaf osmolality might imply the opposite [30,31]. Salinity also reduces CO_2_ assimilation, due to stomata closure [32]. The toxic effect of salt exposure includes chlorophyll degradation due to chloride (Cl) accumulation in the chloroplasts and leaf necrosis due to an increase in the sodium:potassium (Na:K) ratio [33,34,35]. These effects may also decrease the plant’s photosynthetic rates and consequently its reproduction and yield.

The presented work was conducted in a fruit-bearing avocado orchard irrigated with high salinity water (280–300 mg Cl/L^−1^, electrical conductivity (EC) 1.4–1.5 dS/m) for two consecutive years. We used a single scion—‘Hass’—grafted on 20 different rootstocks, which were previously characterized as relatively tolerant to several stresses [12]. The divergent sensitivity of the rootstocks to salt stress was evaluated by several plant stress indicators, together with vegetation and reproduction measurements. The extensive rootstock collection, with 25 repetitions each, provided a unique opportunity to study the response of mature avocado trees thoroughly and to provide consequent applicable recommendations.

## 2. Materials and Methods

### 2.1. Experimental Site and Plant Material

The research was conducted at the Gilat Research Center, Israel (31°20′08.6′′ N 34°39′57.0′′ E). Twenty seedling and vegetatively cloned (VC) rootstocks were grafted in 2011 with ‘Hass’ scions and planted in 2013. The rootstocks represent a wide range of genetic backgrounds, propagation methods, and horticultural characteristics (Table 1). Each rootstock had 25 trees as repetitions, in groups of five trees each in five plots placed randomly in the orchard (Figure S1). Each plot contained, aside from the five ‘Hass’ trees, one ‘Ettinger’ tree grafted onto the same rootstock, as a pollenizer. All trees were pruned uniformly once a year, after harvest. The soil was characterized as sandy loam, with 11.5% calcium carbonate. In December 2018, prior to the exposure to salinity, soil EC and pH in saturated paste extract were 0.73 dS/m and 7.79, respectively. Soil Na and Cl concentrations in the saturated paste extract were 22.27 and 17.62 mg/L, respectively, and soil adsorption ratio (SAR) was 0.9. The orchard was drip-irrigated and fertigated according to local commercial recommendations with liquid fertilizer (Shefer^TM^+3, Fertilizers & Chemicals Ltd., Kiryat Ata, Israel) twice a week, from March to October. The fertilizer solution contained 7% nitrogen (N), 2% P_2_O_5_ and 7% K_2_O, 300 mg/kg iron (Fe), 150 mg/kg manganese (Mn), 75 mg/kg zinc (Zn), 11 mg/kg copper (Cu), and 8 mg/kg molybdenum (Mo). Annual irrigation was 14,508 m3/ha, and annual liquid fertilization was 2940 kg/ha.

In March 2019, NaCl was added to the fertigation solution to reach 280–300 mg Cl/L, with EC of 1.4–1.5 dS/m. This level of salinity is considered harmful but not lethal to avocado [36,37]. A saturated paste extract of the soil in December 2019 had an EC of 1.18 dS/m and pH of 7.32, 70.09 mg/L Cl, 86.82 mg/L Na, and a SAR of 3.81.

During May 2020, extreme weather caused severe burning damage to the edge trees of the orchard. Hence, the following tests did not include those damaged trees.

Throughout the manuscript, we use the rootstock names when referring to either roots, trunks, or leaves. However, the leaves and the upper part of the trunks are genetically identical and belong to the ‘Hass’ scions grafted onto the rootstocks. Using the rootstock names is for distinguishing purposes.

### 2.2. Mineral Analysis, Osmolality, and Trichoms

**Figure 1:**
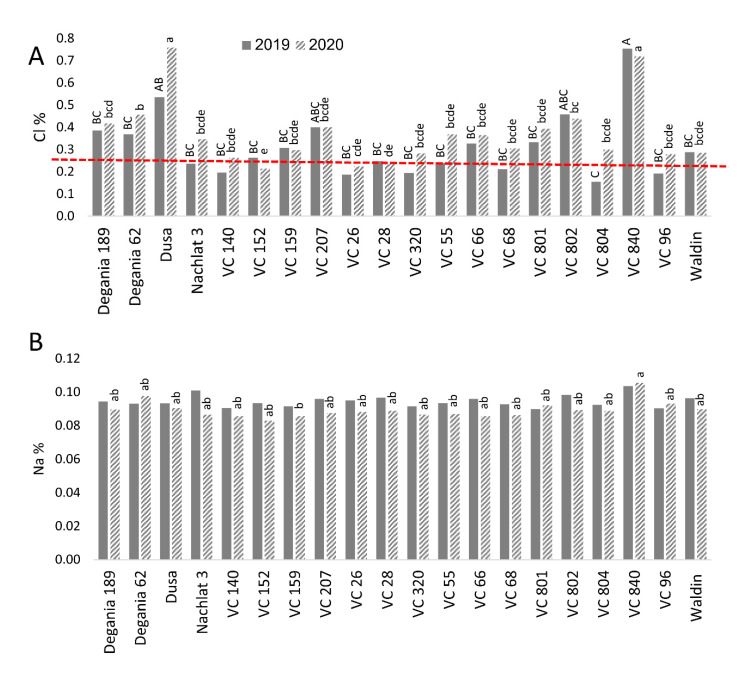
Salt content in leaves of ‘Hass’ avocado trees grafted onto various rootstocks. (**A**): chlorides. (**B**): sodium. Different letters (upper case for 2019 and lower case for 2020) represent significant (*p* ≤ 0.05) differences between rootstocks. The dashed line represents the avocado leaf Cl upper standard suggested by the University of California, above which is considered harmful to the trees. SD values are presented in Table S1.

Diagnostic leaves (youngest fully expanded leaves) were sampled from the trees in November 2019 and 2020. Leaves were dried at 60–70 °C for 48 h, ground and minerals were extracted in water (0.1 g dry matter in 10 mL deionized water). Chloride concentration in the extract was determined using an MKII chloride analyzer 926 (Sherwood) and Na concentration was determined by an atomic absorption spectrometer (Analyst 200, PerkinElmer, Waltham, MA, USA) (Figure 1).

Diagnostic leaves (three leaves per tree) of all rootstocks were sampled on August 2020 and frozen immediately in liquid N. Cell sap was extracted by centrifuging the thawed samples for 10 min at 12,000 rpm. Sap osmolality (mol kg^−1^) was measured using an osmometer (Vapro-5600, Wescor, South Logan, Utah, USA). Diagnostic leaves of all rootstocks were sampled (10 leaves for each rootstock) during August 2020 and observed under florescence stereoscope (Nikon SMZ25, Nikon, Tokyo, Japan). Trichomes were counted in three fixed areas of 300 µm^2^ at the abaxial side of each leaf, and the images were processed with NIS Elements Br software.

### 2.3. Salt Damage Visual Survey

**Figure 2:**
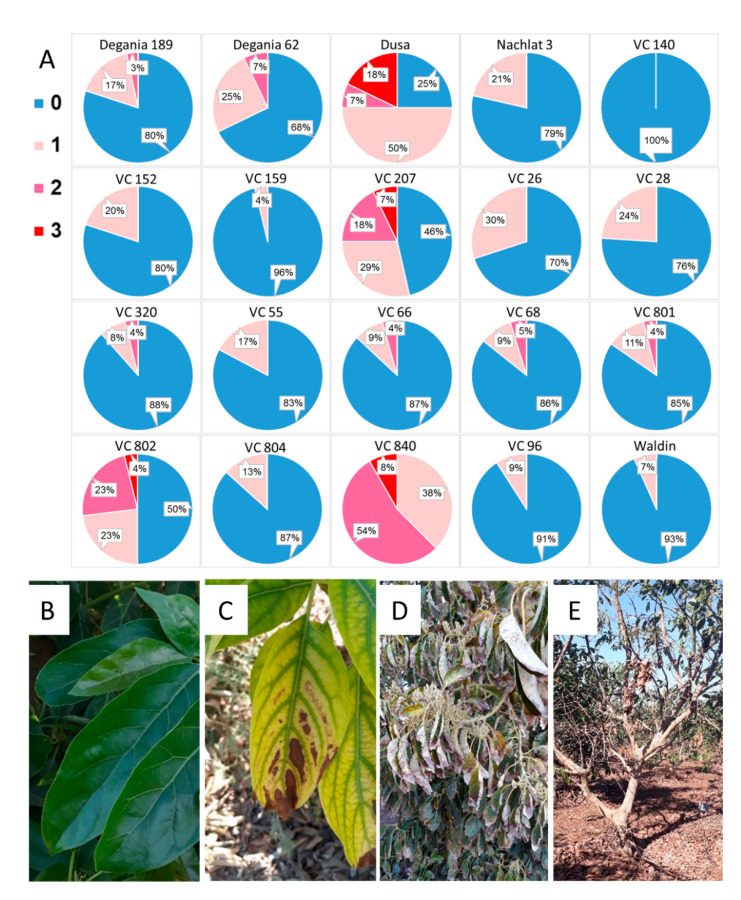
Salt damage in the orchard after a year of irrigation with water high in salt. (**A**): visual survey. (**B**): healthy avocado leaves. (**C**): leaf necrosis. (**D**): severe leaf burns. (**E**): defoliation. In the survey, blue (0) represents no visual salt damage and pink/red shades (1–3) represent salt damage in increasing severity, from light necrosis to tree defoliation.

On April 2020, a survey was taken in the orchard, in which each tree was ranked by its visual salt damage symptoms (leaf necrosis and defoliation) from 0–3. A score of 0 meant “no symptoms”, and scores of 1–3 indicated gradual severity in the symptoms (Figure 2B–E). The score was given to the general performance of the tree, regarding both the young and old leaves.

### 2.4. Vegetation Indices and Photosynthetic Parameters

The trunks of all trees were marked 15 cm above the grafting location. The circumference was measured on December 2018 and then annually after harvest (December) at the mark. Leaf area index (LAI) and photosynthetic active radiation (PAR) were measured on a bright day at noon, in August 2020, by a portable ceptometer (AccuPAR LP-80).

In September 2020, 18 months after initiation of the salinity treatment, on a bright sunny day, midday CO_2_ assimilation, transpiration, and stomatal conductance were measured. These measurements were performed on new mature leaves by a CIRAS-3 portable photosynthesis system (PP Systems).

### 2.5. Remotely Sensed Vegetation Indices

A drone flight was conducted with a DJI Matrice 600 pro (DJI, Shenzhen, China) equipped with a MicaSense RedEdge-MX multispectral camera (MicaSense, Seattle, WA, USA) facing nadir. The Rededge-MX has five sensors (blue: 465–485 nm, green: 550–570 nm, red: 663–673 nm, red edge: 712–722 nm, and near infrared: 820–860 nm). The drone was flown at 12:00 noon to reduce the shadows of trees in the canopy. Flight path was planned and executed as a grid mission with the PIX4D capture 4.10.0(11) application (Pix4D SA; Lausanne, Switzerland) installed on an Apple IPAD. The altitude of the flight was 30 m above ground level, with a front and side overlap of 80%, and the corresponding ground sampling distance (GSD) was 2.08 cm/px. Calibration images of the MicaSense calibrated reflectance panel were acquired before and after the flight to ensure image quality. In addition, 6 ground control points (GCP) were placed on the edges and 1 GCP placed inside the orchard. The coordinates of the GCP were measured using real-time kinematic GPS (Spectra Precision SP60 receiver, Trimble) to increase the accuracy of the image photogrammetric processing. The photogrammetric processing was carried out using the Agisoft Metashape professional 1.6.5 software package to produce an orthophoto and a digital surface model (DSM). Image analysis was performed using ArcGIS 10.8,1 software (ESRI, Ltd., Novi Beograd, Serbia). Plant pixels were identified and extracted by a manual thresholds setting, based on DSM, which represents the height of the canopy, and the green and NIR bands often used to differentiate between soil and vegetation pixels. Following initial extraction of vegetation pixels, the normalized difference red edge (NDRE) vegetation spectral index (Potgieter et al. 2017) was calculated using:

Equation (1):

NDRE = (NIR − red edge)/(NIR + red edge)(1)

NDRE is similar to the common NDVI (normalized difference vegetation index), yet instead of using the red band, it uses the red-edge band. NDRE is considered a better marker of plant conditions when dealing with mature trees, as red-edge light can pass through the leaves far deeper than the red light. Moreover, NDVI often becomes inaccurate (saturated) after plants accumulate their maximum amount of chlorophyll content (Becker et al. 2020).

Avocado leaf pixels were further identified and extracted by a manual threshold setting of NDRE > 0.3. Using a GIS layer of 1.2 m circle polygons for each tree, a mean NDRE value in each polygon was calculated using the tool Zonal Statistics from the Spatial Analyst Toolbox in ArcGIS.

### 2.6. Productivity

Fruits were harvested and weighed separately per tree during the 2019 and 2020 seasons. To calculate the fruit number per tree, a random ten-fruit sample from each tree was weighed.

### 2.7. Stem Water Potential (SWP)

Mature leaves were enclosed in aluminum foil lined sealable bags for two hours before measurement. SWP was measured in a Scholander-type pressure chamber (MRC, Israel) according to [38]. In two rootstocks (VC840 and VC152), SWP was measured a day before the initiation of salinity treatment (March 2019), and four more times at 2-week intervals.

### 2.8. Susceptibility Rating

On each index that was tested in this work (Cl and Na leaf concentrations, osmolality, visual damage, trunk circumference, LAI, CO_2_ assimilation, stomatal conductance, NDRE, trichomes density, and yield), the rootstocks were ranked from 1 to 20, according to their relative salt sensitivity (20—the most susceptible rootstock, 1—the most tolerant one). The average indices’ score of each rootstock was calculated to obtain the final rating.

### 2.9. Statistical Analysis

JMP^®^14.0.0 software (SAS Institute Inc., Cary, North Carolina, USA) was used to carry out ANOVA and correlation analysis. The Tukey–Kramer test was used to estimate the differences between the rootstocks at *p* ≤ 0.05.

## 3. Results

Leaf concentrations of Cl and Na were compared with the toxic thresholds for avocado suggested by the University of California (http://ucavo.ucr.edu/General/LeafAnalysis, accessed on 10 August 2021). Cl leaf concentration measured in most rootstocks was higher than the upper threshold of 0.25% (Figure 1A). However, in some rootstocks, this was the case only in 2020. The highest Cl levels in 2020 were found in Dusa and VC 840 (0.76% and 0.72%, respectively), while the lowest Cl concentration was measured in VC 152 leaves (0.21%). Na leaf concentration was not affected significantly by the rootstocks in 2019 (Figure 1B). However, in 2020 the highest level of leaf Na was found in VC 840 and the lowest in VC 159. No rootstock reached the upper threshold of 0.25%.

After a year of irrigation with water high in salt, salinity damage was observed in some specific rootstocks in the orchard, including leaf necrosis and defoliation (Figure 2). The rootstocks that showed the highest visual salt damage were VC 840, Dusa, VC 207, and VC 802.

**Figure 3:**
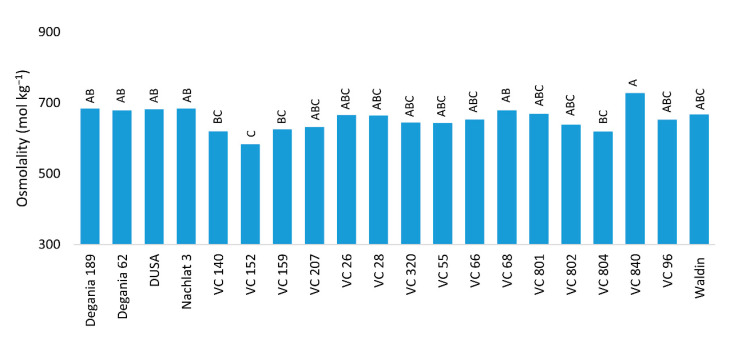
Leaf osmolality of ‘Hass’ avocado trees grafted onto various rootstocks, after 18 months of salinity exposure. Different letters represent significant (*p* ≤ 0.05) differences between rootstocks. SD values are presented in Table S1.

The highest leaf osmolality (Figure 3) was measured in VC 840’s leaves: 728 mol kg^−1^. The lowest osmolality was found in VC 152’s leaves: 583 mol kg^−1^.

**Figure 4:**
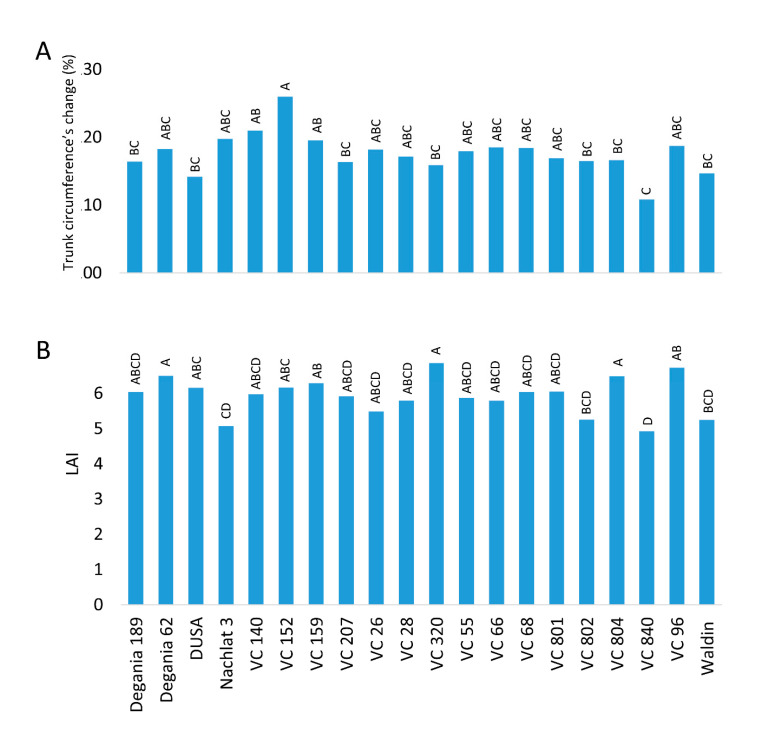
Vegetation indices of ‘Hass’ avocado trees grafted onto various rootstocks. (**A**): the relative change in trunk circumference from 2018 to 2020 (under salinity conditions). (**B**): leaf area index (LAI) measured in August 2020. Different letters represent significant (*p* ≤ 0.05) differences between rootstocks. SD values are presented in Table S1.

The average biannual increase in trunk circumference for all rootstocks was nearly 17% (Figure 4A). VC 152 grew the most, with an increase of 26%, while VC 840’s increase was the lowest—only 11%—and Dusa increased 14%. The highest LAI was found in VC 320, Degania 62, and VC 804 (Figure 4B). The lowest LAI was measured in VC 840 and Nachlat 3.

**Figure 5:**
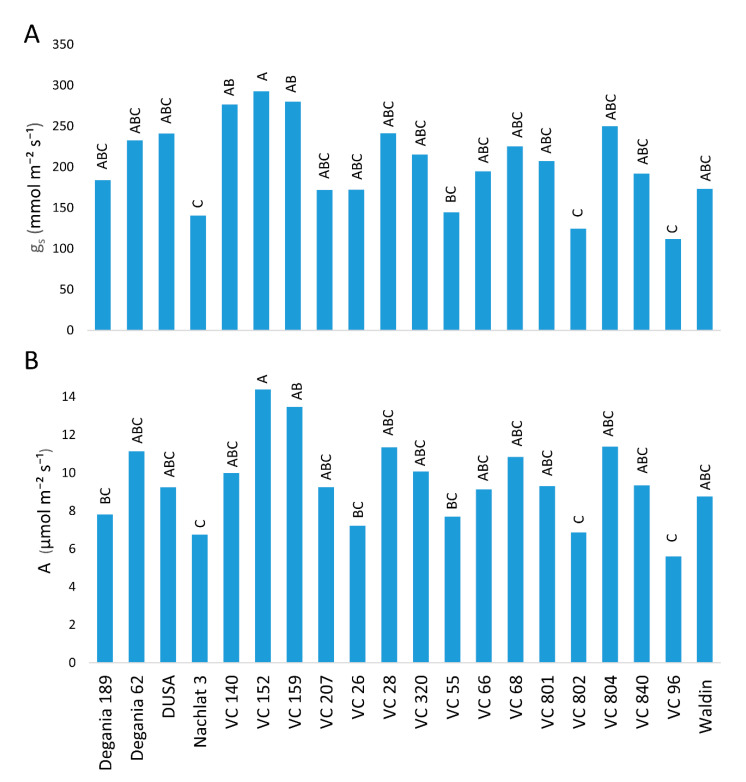
Stomatal conductance (**A**) and CO_2_ assimilation (**B**) of ‘Hass’ avocado trees grafted onto various rootstocks, after 18 months of salinity exposure. Different letters represent significant (*p* ≤ 0.05) differences between rootstocks. SD values are presented in Table S1.

Photosynthesis rates were affected by the rootstock (Figure 5). The highest stomatal conductance (gs) was measured in leaves of VC 152 (Figure 5A). The lowest gs values were measured in Nachlat 3, VC 802, and VC 96 were nearly half the value of highest. Similar results were found regarding CO_2_ assimilation (Figure 5B).

**Figure 6:**
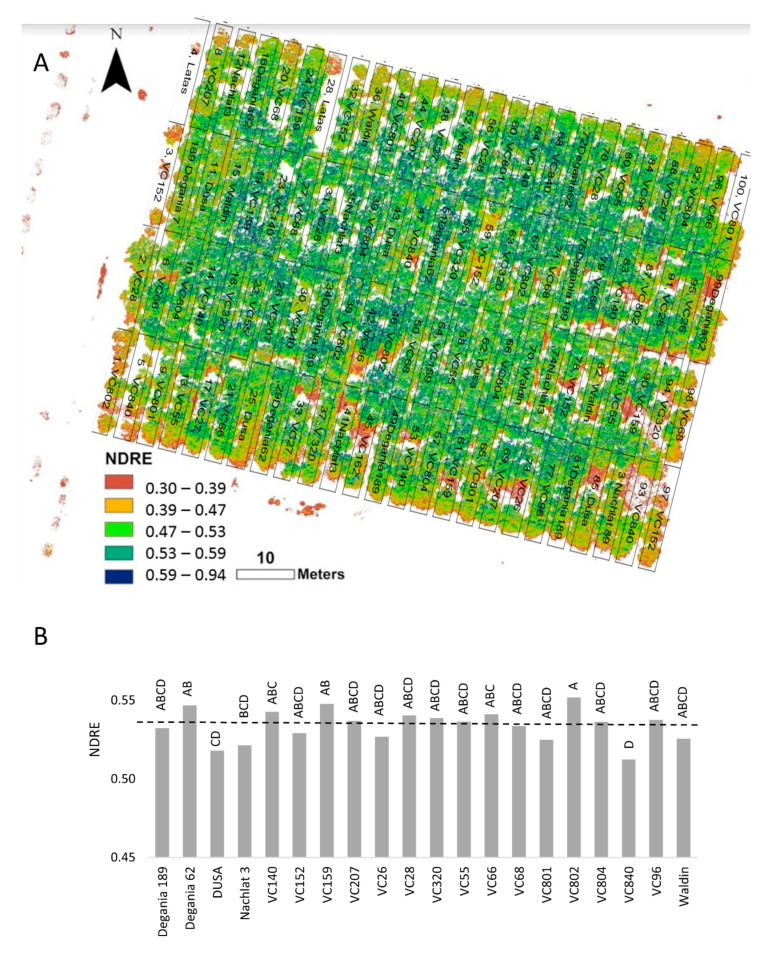
Normalized difference red edge (NDRE) index of ‘Hass’ avocado trees grafted onto various rootstocks, after 18 months of salinity exposure. (**A**): NDRE mosaic visualization over the orchard map. (**B**): average NDRE values according to rootstock. Different letters represent significant (*p* ≤ 0.05) differences between rootstocks. The dashed line represents the 0.53 NDRE value, above which plants are considered non-stressed (see https://micasense.com/what-is-ndre, accessed on 10 August 2021). SD values are presented in Table S1.

In the NDRE mosaic (Figure 6A), red represents bare soil, yellow to pale green indicates stressed plants, and dark green to blue shows vigorous canopy (Figure 6A) (see https://micasense.com/what-is-ndre, accessed on 10 August 2021). We found the highest NDRE values in rootstocks VC 802, Degania 62, and VC 159, and the lowest in VC 840 and Dusa (Figure 6B).

**Figure 7:**
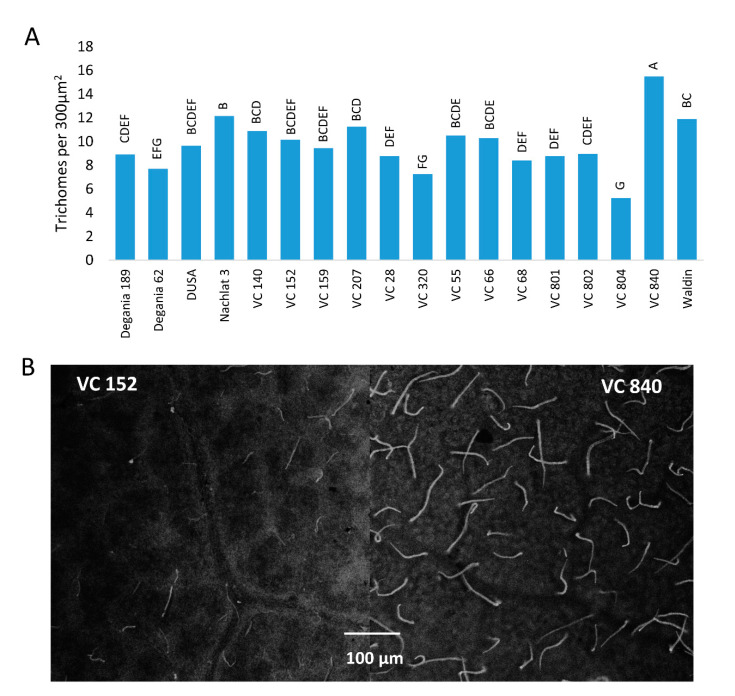
Trichome density on the abaxial side of ‘Hass’ avocado leaves. (**A**): the effect of rootstocks on trichome density after 18 months of salinity exposure. (**B**): live avocado leaves of VC 152 and VC 840 under a stereoscope. Different letters represent significant (*p* ≤ 0.05) differences between rootstocks. SD values are presented in Table S1.

Trichome density was significantly different between ‘Hass’ leaves that were grafted on different rootstocks (Figure 7). The highest quantity of trichomes per 300 µm^2^ was measured in VC 840 leaves (15.5) and the lowest in VC 804 (5.25).

**Figure 8:**
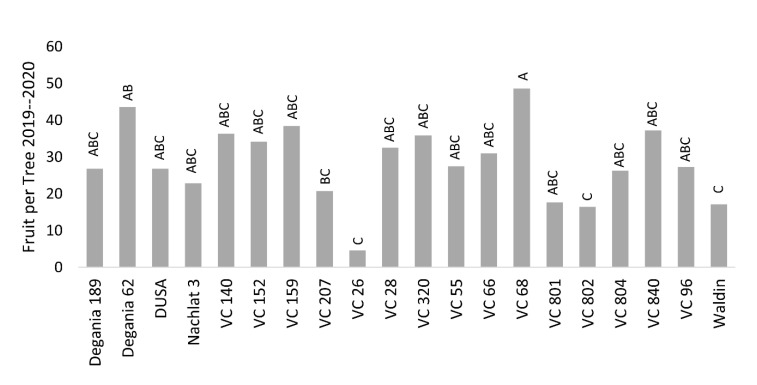
Biannual yield (average number of fruit per tree) of ‘Hass’ avocado trees grafted onto various rootstocks, under salinity exposure. Different letters represent significant (*p* ≤ 0.05) differences between rootstocks. SD values are presented in Table S1.

As avocado trees exhibit alternate bearing cycles, we chose to present the average biannual yield of 2019–2020 (under salinity conditions). We found significant differences between the yields of the rootstocks (Figure 8). The highest yields were those of VC 68 and Degania 62, and the lowest were recorded in VC 26, VC 802, VC 801, and Waldin.

Correlation analyses between the different indices of all rootstocks revealed a significant affinity between the canopy volume and LAI, PAR, yield and the relative change in trunk circumference (Table 2). The photosynthesis indices (gs, A, and E) were highly correlated with each other. The transpiration was also correlated with LAI and PAR. Cl leaf concentration was significantly correlated with LAI, leaf osmolality, trichomes number and the change in trunk circumference.

**Figure 9:**
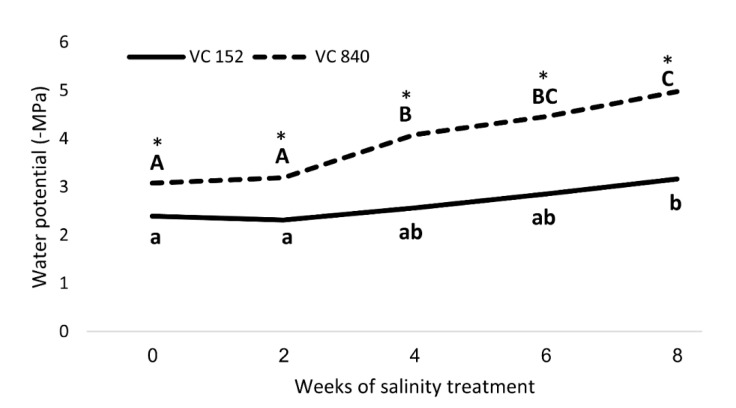
Stem water potential in ‘Hass’ avocado trees grafted onto either VC152 or VC840 rootstocks along eight weeks of salinity treatment. * represents a significant difference (*p* ≤ 0.05) between the rootstocks. Different letters represent significant differences between measurement dates in a specific rootstock.

The two rootstocks that exhibited the highest and lowest leaf Cl—VC 840 and VC 152, respectively—were chosen for an in-depth investigation of their response to salt in terms of water stress. These rootstocks had a significant difference in leaf Cl and SWP even before salinity exposure, but the response to stress, as expressed in SWP, was faster and stronger in VC 840, compared with VC 152 (Figure 9).

For each salt-related index tested in this work, rootstocks were scored progressively from 1 to 20, according to their measured performance; 1—the least salt sensitive rootstock, and 20—the most sensitive. We calculated the overall score of the rootstocks (Table 3) and the results demonstrate the divergent susceptibility of avocado rootstocks to salt. The most susceptible rootstocks were VC840, Dusa, and VC802. The least susceptible were VC159, VC140, and VC152.

## 4. Discussion and Conclusions

The fact that leaf Cl concentrations are influenced by rootstocks has been reported for many crops, among them grapevine [39], pomegranate [40], citrus [41], and avocado [18]. This phenomenon is likely to stem from divergent levels of root Cl absorption and exclusion, as well as transport mechanisms [42,43,44]. In our experiment, the rootstocks that exhibited the highest leaf Cl concentration (VC 840 and Dusa, Figure 1) were both of a Mexican genetic background, which is characterized by this trait [17,45,46]. The combination of ‘Hass’ scion with a Mexican rootstock was found to be extremely salt-sensitive, with significant damages when the EC is higher than 0.6 dS/m [47].

Dusa, the second most susceptible rootstock in our orchard, was reported as a top-producer under salinity conditions similar to ours (EC = 1.5 dS/m) in a previous study, which compared 13 avocado rootstocks in California [48]. However, this result changed after the first year of their experiment. Considering the fact that most of the trees in [48] did not survive the experiment, we suggest that the rootstocks that were included in the experiment were relatively salt-sensitive, while our rootstock collection comprised a wide range of responses to salinity, including various levels of tolerance, as reported in [12,16,49,50]. However, in our study, Dusa leaves accumulated more Cl ions in the second year of the experiment, compared with the first one (Figure 1), which suggests a gradual response to salt exposure, similarly to [48].

Visual salt damage was most severe in the rootstocks with a Mexican background (VC 840, Dusa, and VC 207), but also in a West Indian rootstock—VC 802 (Figure 2). Leaf necrosis and defoliation are known manifestations of the toxic effect of Cl, as was reported, among others, for citrus [51], and poplar [52]. A common indication of ion accumulation in the leaves is the osmolality [53], which was also highest in VC 840 (Figure 3). Leaf osmolality increased under NaCl stress in grapevine [54] and almonds [55], where Cl ions were found to account for most of it.

In accordance with the known limitations of vegetative growth due to salinity [56], we observed relative differences in LAI and trunk circumference between the rootstocks in the orchard. VC 840 was the most effected rootstock for both variables (Figure 4). VC 152, which was ranked highest in trunk growth, accumulated the least amount of Cl in the leaves and exhibited the lowest leaf osmolality. These results strengthen the effect of stress on plant vigor, and the rootstock’s major influence over both salt-susceptibility and vegetative growth.

Salt stress may limit the photosynthetic capacity of crop plants and harm their growth and development [57]. High leaf concentrations of Na are known to reduce stomatal conductance [58]. High leaf concentrations of Cl are known to damage photosynthesis via chlorophyll degradation [59]. Chlorophyll degradation and low stomatal conductance reduce CO_2_ assimilation [60]. This is in line with our results, where VC 152, which had the lowest salt leaf concentrations, showed the highest photosynthesis rates (Figure 5). A recent research focused on the most salt-tolerant rootstocks from [48]—R0.05, Dusa, and PP40—and investigated their development, photosynthesis rates, and physiological traits [61]. Similar to our results, they found that salinity decreased photosynthesis and impaired plant performance.

Spectral reflectance was previously used to measure salinity damage in sunflowers [62] and several landscape species [63]. In avocado orchards, spectral tools were found to be accurate in evaluating the vegetative status [64]. NDRE was chosen for our experiment due to its high sensitivity compared with NDVI [65]. NDRE values supported our other findings of VC 840 and Dusa as the most salt-susceptible rootstocks in the orchard (Figure 6).

The highest trichome density was found in the leaves of VC 840 (Figure 7) and the lowest in VC 804, which exhibited high LAI (Figure 4) and the lowest leaf Cl in 2019 (Figure 1). It is noteworthy that LAI and trichome density were negatively associated in other rootstocks as well, although we did not find a significant correlation between these indices (Table 2). This finding is novel for avocado. As trichome density was found to increase under salinity in several other plants [66,67,68], we suggest our original finding as an additional indicator for salt susceptibility in avocado rootstocks. The physiological or developmental mechanism that correlates trichomes with salinity exposure is a subject for additional research.

Salt stress is known to negatively affect plant productivity [57]. Reproductive development can be quantified in several phenological stages—flowering, fruit set, fruit growth, and the resulted yield. In this work, we chose the final number of fruit per tree (FTP) as the objective, but did not find strong associations between the stress indications and the yields (Table 2). The highest FTP was found for VC 68 and Degania 62, and the lowest were recorded for VC 26, VC 802, VC 801, and Waldin (Figure 8). However, VC 802 had a relatively high leaf Cl concentration and low photosynthesis rates, and Degania 62 exhibited high LAI and low trichome density. We assume that in an alternate-bearing tree like avocado, the salinity effect over the yield will be strengthened as the salt treatment continues for years.

After considering all the results, we focused on two rootstocks with opposing response to salt—VC 840 (most susceptible) and VC 152 (least susceptible). Measurements of SWP revealed a significant difference between these rootstocks (Figure 9): VC 840’s SWP was more negative even before the initiation of salt treatment, and the trees exhibited stress indication weeks before the VC 152 trees did. VC 840 stress reaction was more intensive than that of VC 152, as the relative change in SWP due to saline irrigation was stronger in VC 840 (Figure 9). This result, which suggests that differences in salt sensitivity can be predicted even before salinity exposure, requires further validation and extension.

To conclude, salinity is one of the most limiting factors in avocado cultivation, and finding a tolerant rootstock is a challenge for the global avocado industry. There are hundreds of known avocado rootstocks from which commercial nurseries choose their grafting material. Our work was based on ongoing Israeli research programs, representing a wide range of rootstocks with divergent characteristics and relatively high tolerance to biotic and abiotic stresses. The presented work contributes to the global avocado database by comparing the physiological response of 20 commercial rootstocks to salt exposure. In addition to our specific results, we believe that the methodologies that were used in the study can provide an applicative and desirable tool for avocado growers and researchers in areas concerned with soil salinity, particularly when induced through low-quality irrigation water.

## Figures and Tables

**Table 1:** Rootstocks evaluated in the experiment.

Rootstock	Origin	Propagation Method
Degania 189	WI	Seedling
Degania 62	WI	Seedling
Dusa	Mex × Gu	VC
Nachlat 3	WI	Seedling
VC 140	WI	VC
VC 152	WI	VC
VC 159	WI	VC
VC 207	Mex × WI	VC
VC 26	WI	VC
VC 28	WI	VC
VC 320	WI	VC
VC 55	WI	VC
VC 66	WI	VC
VC 68	WI	VC
VC 801	WI	VC
VC 802	WI	VC
VC 804	WI	VC
VC 840	Mex	VC
VC 96	WI	VC
Waldin	WI	VC

WI—West Indian; Mex—Mexican; Gu—Guatemalan; and VC—vegetatively cloned.

**Table 2:** Correlation probability between the developmental and physiological indices that were tested among the rootstocks. g_s_: stomatal conductance. A: CO_2_ assimilation. E: transpiration. LAI: leaf area index. PAR: photosynthetic active radiation. Osmol: leaf osmolality. FPT: fruit per tree. Trich: trichomes. TC: trunk circumference. Bold font represents significant (*p* ≤ 0.05) correlation between indices.

	Canopy Vol.	g_s_	A	E	LAI	PAR	Osmol.	Yield 19–20 (FPT)	Trich	Cl (%)
g_s_	0.7717									
A	0.2217	**<0.0001**								
E	0.1758	**<0.0001**	**<0.0001**							
LAI	**<0.0001**	0.0522	0.2099	**0.0043**						
PAR	**<0.0001**	0.9557	0.8939	**0.0452**	**<0.0001**					
Osmolality	0.3125	0.7451	0.8919	0.5665	0.8503	0.3706				
Yield 19–20 (FPT)	**0.0163**	0.2960	0.4726	0.5119	**0.0012**	0.7984	0.8084			
Trichomes	0.7245	0.2420	0.9788	0.0693	0.3993	**0.0338**	0.2112	0.7814		
Cl (%)	0.9285	0.4523	0.4686	0.7877	**0.0108**	0.3409	**0.0131**	0.0657	**0.0002**	
Change in TC (%)	**0.0050**	0.3234	0.0649	0.2748	0.3633	0.6928	**0.0048**	0.3140	0.8209	**0.0304**

**Table 3:** Salt susceptibility rating of avocado rootstocks under EC 1.4–1.5 dS/m.

Rootstock	Final Score	
VC 840	16.8	Least susceptible ←--------------------------→ Most susceptible
Dusa	15.2	
VC 802	14.8	
Degania 189	12.8	
VC 801	12.7	
VC 207	12.6	
Waldin	12.5	
Nachlat 3	12.2	
VC 26	11.1	
VC 55	11.1	
VC 66	10.1	
VC 96	9.4	
VC 28	9.4	
Degania 62	9.3	
VC 68	8.5	
VC 320	7.1	
VC 804	6.4	
VC 152	6.0	
VC 140	5.8	
VC 159	4.8	

## Supplementary Materials

The following are available online at https://www.mdpi.com/article/10.3390/plants10081672/s1, Figure S1: The orchard map, Table S1: SD values for figures 1, 3, 4, 5, 6, 7, and 8.
