# Supplementary material for: Rootstock-Dependent Response of Hass Avocado to Salt Stress
Source: Plants (Basel). 2021 Aug 13;10(8):1672. doi: 10.3390/plants10081672 (PMC8399844; doi:10.3390/plants10081672)
Supplement: Supplementary file 1 [file plants-10-01672-s001.zip › plants-1339252-supplementary/Figure S1.pdf]

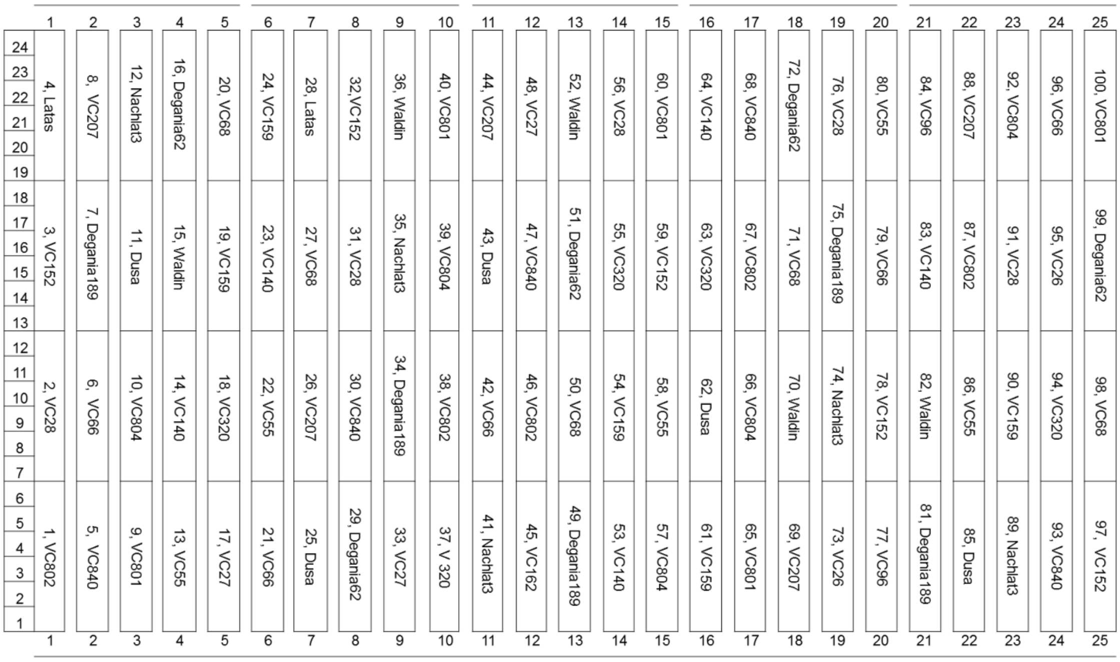

Figure S1. The orchard map.
