# Supplementary material for: Rootstock-Dependent Response of Hass Avocado to Salt Stress
Source: Plants (Basel). 2021 Aug 13;10(8):1672. doi: 10.3390/plants10081672 (PMC8399844; doi:10.3390/plants10081672)
Supplement: Supplementary file 1 [file plants-10-01672-s001.zip › plants-1339252-supplementary/Table S1.pdf]

Table S1. SD values for figures 1, 3, 4, 5, 6, 7, and 8.

# Figure	1				3	4		5		6	7	8
Rootstock	Cl 19	Cl 20	Na 19	Na 20	Osmolality	Trunk circum.	LAI	g_s	A	NDRE	Trichome density	FPT
Degania 189	0.15	0.06	0.01	0.01	29.47	3.74	1.06	42.15	3.33	0.02	3.06	19.09
Degania 62	0.33	0.12	0.01	0.02	75.67	5.99	0.74	98.42	3.47	0.02	2.58	33.17
Dusa	0.04	0.11	0.01	0.01	55.73	4.76	1.31	135.56	4.92	0.02	4.99	13.68
Nachlat 3	0.11	0.04	0.00	0.01	85.62	8.95	0.91	55.98	1.60	0.02	2.98	29.75
VC 140	0.16	0.06	0.01	0.00	64.71	4.72	0.67	89.51	5.48	0.03	3.34	28.60
VC 152	0.09	0.04	0.01	0.01	45.52	4.14	1.10	40.72	2.63	0.02	2.25	32.90
VC 159	0.17	0.14	0.00	0.01	60.17	4.42	0.99	95.82	4.28	0.02	2.39	23.51
VC 207	0.12	0.07	0.01	0.01	43.47	4.13	0.52	71.65	3.69	0.03	2.51	19.42
VC 26	0.19	0.18	0.01	0.01	36.66	4.38	0.98	66.24	1.94	0.02	NA	4.41
VC 28	0.08	0.10	0.01	0.00	64.86	6.14	1.29	61.20	2.84	0.02	2.48	47.37
VC 320	0.11	0.12	0.00	0.00	51.17	4.70	0.90	66.87	3.63	0.02	1.86	27.72
VC 55	0.04	0.07	0.01	0.00	28.76	6.50	0.89	70.93	5.56	0.01	1.88	18.06
VC 66	0.01	0.02	0.01	0.00	47.63	5.18	0.95	63.72	3.32	0.02	2.52	19.06
VC 68	0.04	0.11	0.01	0.01	65.21	4.95	0.65	158.31	4.17	0.02	2.04	45.00
VC 801	0.10	0.07	0.01	0.00	40.79	4.57	0.52	59.36	2.51	0.03	2.59	16.10
VC 802	0.28	0.05	0.01	0.01	40.41	6.75	1.41	44.72	3.23	0.02	2.54	7.79
VC 804	0.09	0.08	0.01	0.00	49.00	5.20	0.80	118.18	4.84	0.02	1.55	16.51
VC 840	0.25	0.25	0.01	0.03	91.68	5.29	1.53	79.12	3.59	0.05	3.05	37.73
VC 96	0.30	0.15	0.01	0.00	81.16	2.34	0.28	70.69	3.40	0.01	NA	16.74
Waldin	0.08	0.03	0.01	0.00	56.86	7.65	1.08	70.57	2.43	0.02	2.88	18.65
